# Behrens' *General Botany*

**Published:** 1885-06

**Authors:** 


*Text-Book of General Botany.* By Dr. W. J. BEHRENS. Translation from the second German Edition, revised by P. GEDDES. Pp. 374. Edinburgh: Y. J. PENTLAND. 1885.

THE excellence of Dr. Behrens' Text-Book on Botany is well known. We have only to say a word in praise of the manner in which the translator (Miss Harris Smith) has done her work, and of the style in which the publisher has produced it. The clear, simple language ; the abundant high-class illustrations ; the accurate divisions and sub-divisions ; and, above all, the moderate bulk of the work, are sure to make it a favourite text-book with English students. A special feature in the work is the section on fertilisation, in which an account, more perfect than in any other work with which we are acquainted, is given of the transference of pollen by insects. We have no hesitation in stating our belief that this is the best Botanical Text-Book for medical students in the English language.

